# Distinct varieties of aesthetic chills in response to multimedia

**DOI:** 10.1371/journal.pone.0224974

**Published:** 2019-11-14

**Authors:** Scott Bannister

**Affiliations:** Department of Music, Durham University, Durham, County Durham, England, United Kingdom; Victoria Univ Wellington, NEW ZEALAND

## Abstract

The experience of aesthetic chills, often defined as a subjective response accompanied by goosebumps, shivers and tingling sensations, is a phenomenon often utilized to indicate moments of peak pleasure and emotional arousal in psychological research. However, little is currently understood about how to conceptualize the experience, particularly in terms of whether chills are general markers of intense pleasure and emotion, or instead a collection of distinct phenomenological experiences. To address this, a web-study was designed using images, videos, music videos, texts and music excerpts (from both an online forum dedicated to chills-eliciting stimuli and previous musical chills study), to explore variations across chills experience in terms of bodily and emotional responses reported. Results suggest that across participants (N = 179), three distinct chills categories could be identified: *warm chills* (chills co-occurring with smiling, warmth, feeling relaxed, stimulated and happy), *cold chills* (chills co-occurring with frowning, cold, sadness and anger), and *moving chills* (chills co-occurring with tears, feeling a lump in the throat, emotional intensity, and feelings of affection, tenderness and being moved). Warm chills were linked to stimuli expressing social communion and love; cold chills were elicited by stimuli portraying entities in distress, and support from one to another; moving chills were elicited by most stimuli, but their incidence were also predicted by ratings of trait empathy. Findings are discussed in terms of being moved, the importance of differing induction mechanisms such as shared experience and empathic concern, and the implications of distinct chills categories for both individual differences and inconsistencies in the existing aesthetic chills literature.

## Introduction

The experience of aesthetic chills is often characterized as a subjective response accompanied by either goosebumps, shivers or more elusive tingling sensations. The response has been a useful indicator of strong emotional experiences in experimental settings, given a correspondence between physiological activity (e.g. skin conductance [1], pupil dilation [2], and goosebumps [3, 4]), and subjective feeling components of emotion [5, 6]. Chills have further been linked to pleasure and reward systems in the brain when listening to music [7–9]. Importantly, whilst shivers and tingling are often included in working definitions of chills, only the goosebumps response appears to have been objectively captured during engagements with music, film and poetry [1, 3, 4].

Chills have historically attracted notable attention in the domain of music, with correlations found between the response and certain musical features, such as dynamic or textural changes [5, 10, 11], solo and accompaniment interactions [12], lyrics and the human voice [13], and unexpected harmonic changes [11]; further psychoacoustic considerations have linked chills with loudness and auditory roughness [14]. Experiences of chills are not restricted to music however; Goldstein [15] noted that common elicitors may be perceived beauty in nature and art, scenes in a movie, play or book, and physical contact with another person. Indeed, recent research has assessed the experience in other domains, such as film, poetry and religious contexts. With film, Benedek and Kaernbach [3] suggested that chills were effectively elicited by audio clips from films; these audio clips included an astronaut saying goodbye to his daughter before a meaningful sacrifice, and a leader of suppressed people encouraging the fight against tyranny. Conversely, Wassiliwizky, Wagner, Jacobsen and Menninghaus [16] utilized film clips portraying reunions, declarations of love, and death of partners or loved ones to elicit chills in participants. Regarding poetry, a recent study reported that chills were mostly linked to specific aspects in a spoken poem, including lines emphasizing communicative or social aspects, for example addressing a lover or other person [4]; other notable structural features linked to chills include cadences and the closure of lines. Finally, recent work noted the experience of chills and tingling sensations in religious contexts [17, 18]; similar contexts and events are also reported in accounts of strong experiences with music [19].

Despite the attention directed towards aesthetic chills, there is no explanation as to how the experience is elicited by wide varieties of features in music, film, poetry and other multimedia items. Consequently, several theories have been developed that, each in isolation, fail to account for the existing findings in the literature, and are difficult to reconcile with each other. Therefore, it is currently unclear as to whether chills reflect a unified psychological construct of peak pleasure, or instead refer to a set of distinct categories of response, varying in terms of experience, elicitors, and individual differences. This possibility of distinct varieties of aesthetic chills serves as the central focus of the current study.

### Theories of aesthetic chills

#### Fear and vigilance

Whilst experimental approaches to identifying the causal processes underlying aesthetic chills are in their infancy [20], various theories have been postulated with regards to how the phenomenon can be elicited by aesthetic stimuli such as music and film. Firstly, chills have been linked to fear and vigilance, given the evolutionary functionality of goosebumps as a threat-signalling response for conspecifics regarding an approaching threat [21]. These ideas appear most applicable to cases of musical chills. Huron [22] suggested that chills elicited through these processes are linked to mechanisms of anticipation and expectancy, with expectancy violations leading to a worst-case scenario fear appraisal, resulting in goosebumps; subsequently, a slower aesthetic appraisal follows, in which the context is understood as ‘safe’ or distanced from pragmatic life concerns [23], resulting in a contrastive valence effect from fear to pleasure. Harmonic expectancy violations have been linked to physiological arousal [24] and perceptual processing [25, 26], and the fear and vigilance account appears to make sense of correlations between chills and dynamic changes or unexpected harmonic changes previously reported [5, 10, 11]. However, if fear and vigilance were central mechanisms of chills, the response should be reasonably predictable, consistent inter- and intra-individually, and prevalent across a population; this is rarely the case, with notable differences in chills reports across individuals [13], and an estimate that chills may not be experienced in roughly half of the general population [27].

#### Being moved and social processes

An alternative account of aesthetic chills is concerned with social processes, referring to theories or constructs such as social separation, being moved, and communal sharing relations; in contrast to fear and vigilance processes, this account may explain associations between chills and lyrics in music [13], films [3], poetry [4] and religious, communal experiences [17, 18]. Previously, Panksepp [10] suggested that chills could be elicited by certain acoustic qualities in a piece that resemble mammalian distress vocalizations, indicating social separation and encouraging reunion by inducing feelings of coldness. These ideas stem from the thermoregulatory role of goosebumps, as opposed to threat-signalling functions, and may be explained by a degree of anatomical and functional overlap of thermoregulatory and social functions in the brain [28]. Some recent evidence supports this proposition, with feelings of physical warmth and social warmth appearing to share similar patterns of neural activity [29]; furthermore, it has been suggested that social exclusion may result in lower levels of skin temperature [30]. The evolutionary origins of this coupling are unclear, but the development may stem from many years of social thermoregulation techniques, or sharing body heat with others to overcome our ineffective methods of self-regulating body temperature [31].

A recent concept linked to these social processes is being moved, a mixed emotional state largely comprised of amalgams of joy and sadness [32, 33]. This enigmatic construct is linked to elicitors such as significant life events, including weddings, funerals and separations [34], reconciliation between two estranged friends and unexpected kind gestures [35], sad music and films [16, 36], and prosocial cues in film and poetry [4, 37]. The experience appears to be pleasurable and desirable [37], and chills have importantly been suggested to be a physiological indicator of the experience [3, 16, 38]. Being moved shares some similarities with elevation, supposedly elicited by unexpected moral virtue and altruism [39], of which goosebumps may also be an indicator [40]; furthermore, nostalgia is similar in that it is often characterized as a mixture of positive and negative feelings [41], and appears to be motivated by social processes [42, 43]. Being moved may also be comparable to the concept labelled kama muta, described as a positive emotional experience with physical concomitants including warmth in the chest, tears, goosebumps, shivers and a lump in the throat [44]. Pertinently, sensations perceived as occurring in the chest have recently been linked to social closeness, longing, love, togetherness, and sadness [45]; furthermore, tears have recently been reported to co-occur with chills in particularly intense emotional experiences [46].

Although being moved is not a theory of chills, a potential underlying mechanism of the experience, and possibly chills, is the sudden intensification of communal sharing relations (CSRs) [44]. Derived from the broader relational model theory [47], CSRs constitute the perception of social equivalence between oneself and another person, character, group, sub-culture, or other holistic levels of identification; this may be embodied in feelings such as patriotism, love, union or connectedness. However, being moved may also be elicited through observing the intensification of CSRs in others, or in the absence of any other person, instead through introspective processes such as episodic memory.

There are two notable examples of how being moved may be elicited through intensifed CSRs. The first is through moments of *shared experience*, referring in the present context to an equivalence of some kind across bodily substances, surfaces or motions [48]. Consider for example wearing a football team’s uniform and colours at a stadium, surrounded by thousands of visually and audibly like-minded individuals, and singing or moving in unison with these other fans; similar scenarios may be found at music concerts, gigs, or raves [49], or in ritualistic circumstances across different cultures [50]. The second example is related to empathy. Trait empathy has been linked to being moved by music [51], intense emotions with sad music [52], differences in psychophysiological responses to opera [53], and a closer coupling between perceived and experienced emotion during music listening [54]. In other work, appreciation of artworks was increased in those with higher scores on an emotional contagion survey [55], and empathic distress was found to increase the enjoyment of tragic films [56]. A pertinent finding is the proposed link between *empathic concern*, a culturally appropriate, incongruent response (i.e. not mirroring the observed emotion), and chills, tears and feeling warmth in one’s own body [57]. A typical eliciting stimulus may express unfortunate circumstances, or portray a person or other in need of help or support [58, 59]. It may be possible that certain empathic processes can elicit a sudden intensified CSR between one person and another entity, or that an observation of interpersonal empathic concern in an external event might similarly elicit experiences of being moved.

### Chills as a variable construct

In summarizing existing research on aesthetic chills, three main conclusions can be made: Firstly, chills can be elicited by a wide variety of stimuli, be these music, video, texts, or images; secondly, chills appear to be elusive experiences, given an apparent lack of stimulus-response patterns in music and emotion research, inconsistency in inter and intra-individual chills experiences, and the estimate that a significant portion of a population rarely experiences chills [27]; finally, there are numerous correlations reported between chills and features of music, film and poetry, and the proposed theories are difficult to reconcile with each other. These conclusions, alongside recent evidence of significant variability in the emotional experiences reported in musical chills [13], suggest that the current conceptualization of aesthetic chills as an experience of peak pleasure is not adequately nuanced or detailed, and that the chills construct may encapsulate numerous distinct experiences; consequently, it is unknown as to whether the current definitional boundaries of the phenomenon are adequate.

The issue of how chills are currently conceptualized can be further understood through a closer look at existing research. Firstly, it has been documented that in relatively ‘safe’ contexts, depending on the stimulus, listeners can also experience negative chills. For example, Halpern, Blake and Hillenbrand [60] exposed participants to various ‘chilling’ sounds, noting that the most unpleasant sounds include scraping on a slate surface, and rubbing two pieces of polystyrene foam together. Similarly, Grewe, Katzur, Kopiez and Altenmüller [61] showed that chills were experienced across different music excerpts, sounds, images, and tactile or gustatory stimulation, and that emotional experiences varied notably across these stimuli, although these differences were independent of sensory modality.

Secondly, in the case of music, it does not appear intuitive to approach sudden dynamic changes or unexpected harmonic changes from the perspective of being moved; likewise, it seems unlikely that moving film clips activate underlying fear or vigilance mechanisms that may be more prevalent in musical chills, although it must be noted that horror or psychological thriller films may be described as ‘chilling’, and are worthy of investigation. In line with this, Pelowski et al. [62] provide a tentative distinction between thrills and chills; thrills may be linked to novelty or new-found insight, involving feelings of tension, awe or sublimity, whereas chills may be related to absorption and being moved, resulting in peak emotional experiences. Elsewhere, Levinson [63] also suggested that there are two types of musical chills responses, either of short duration elicited through timbre and dynamics, or of longer duration induced through melodic, harmonic or rhythmic developments. No research exists that has addressed these possible distinctions across chills responses.

Finally, closer reading into being moved suggests that whilst joy and sadness are key ingredients, there are two main variations of moving experiences, namely being joyfully or sadly moved [16, 33]. Being joyfully moved may be in response to positive events within negative contexts (reunion after a long separation), whereas being sadly moved may be elicited by negative events within positive contexts (self-sacrifice to save one’s family); notably, similar mixed narratives have recently been linked to aesthetic chills responses [64]. Additionally, whilst being moved might be conceptualized as a broad communal sharing emotion [44], there is the complication of first-, second- and third-person CSRs, empathic concern or shared experience, and whether there are phenomenological distinctions derived from these differing induction processes. For example, first-person CSRs linked to memory may elicit feelings of nostalgia and longing; second-person CSRs may be associated with compassion and empathic concern [57]; and third-person CSRs may elicit more varied emotional experiences, depending on the context. Importantly, little research has inquired about the phenomenological distinctions between different states of being moved, and this highlights the important question of whether being moved is a distinct construct, or whether there are several differing components [65], that may have important implications for chills research.

The present complications and difficulties of conceptualizing chills and categorizing the underlying psychological mechanisms have been acknowledged and investigated in a series of studies by Maruskin, Elliot and Thrash [66]. In their work, it was concluded that the chills construct may be split into positive, approach ‘goosetingles’, and negative, avoidance ‘coldshivers’; further findings suggest that goosetingles were correlated with the personality trait of extraversion, and coldshivers with neuroticism. Finally, with regards to elicitors, goosetingles were more strongly linked to aesthetic beauty, although some instances of coldshivers were also reported. Whilst this is important work in understanding aesthetic chills, the picture appears to be more nuanced than that of holistic approach and avoidance behaviour; chills experiences may involve feelings of awe, surprise, tension, pleasure, being moved, elevation, nostalgia, and may also be characterized as positive and desirable, or negative and aversive. In addition, these varied emotional experiences linked to chills may be distinguished by the role of different underlying psychological processes. To re-contextualize and add clarity to existing research on the phenomenon, and to inform associated psychological concepts, these possible variations of the chills response need to be considered.

### Rationale for the current study

If the phenomenon of chills is not a unified construct, then there are far reaching implications for existing research and theory. Firstly, correlational studies of musical chills may have presented misleading conclusions if certain musical features or stimuli are more effective in eliciting certain chills experiences over others that have not been specified; with consistent use of classical music stimuli in musical chills studies, this could result in an over-representation of certain relationships between music and chills, and not others such as lyrics and the human voice [13]. Secondly, should there be significant distinctions between chills responses, there is the issue of individual differences. The general frequency of musical chills has previously been linked to openness to experience [67–69], but individual differences have not been assesed in relation to variations in chills experience, or current theories of the phenomenon. Also, trait empathy has rarely been explicitly considered in relation to chills experiences, yet is associated with the goosebumps response [57] and related affective states such as being moved [51]; there was thus an opportunity for theoretically grounded, novel investigations into individual differences in trait empathy and chills. Finally, if the conceptualization of aesthetic chills experiences is currently unsuitable, then the causal testing of existing theories cannot develop coherently, meaning that certain hypotheses might be disputed or disregarded, mainly due to errors or inconsistencies in defining and understanding what exactly is being investigated.

The main aim of the current study was to empirically investigate the chills phenomenon and possible variations in the experience, using a variety of stimuli in different modalities and of different thematic qualities. If the chills response is linked to different states such as awe, being moved and peak pleasure, variations in the response should be observable in reported subjective feelings attributed to chills. Furthermore, given that chills can coincide with a variety of other physiological activity such as tears [46], and warmth or tension in the chest [40], and with recent work suggesting that different felt emotions might be attributed to different areas of the body [45], meaningful variations in chills experiences may also be captured through reported bodily responses. The main hypotheses of the study can be formally stated as follows:

- H1: Distinct categories of chills experiences would be established, derived from differences in subjective feeling and bodily activity reported.
- H2: The distinct chills categories would be consistently related to certain thematic qualities of the stimuli.
- H3: Trait empathy would be associated with the categories of chills experience.

## Materials and methods

### Design

A web-study was designed to assess the experience of aesthetic chills in response to 15 multimedia stimuli, across five modal categories (images, videos, texts, music, and music performance videos). These stimuli were derived from both a previous musical chills experiment [20], and an online internet forum dedicated to content that elicits what users label frisson, on the social media website Reddit (www.reddit.com/r/frisson). On this forum, users submit a variety of multimedia content that gives them the experience of frisson, and other users can discuss and comment on the content, with each user being able to cast a vote for each item (+1 for good, enjoyable or effective elicitor, and -1 for the opposite); this provides a user-generated hierarchy of effect across the thousands of items uploaded to the forum, with presumably the most effective elicitors of frisson voted to the top of the rankings. The study followed a repeated-measures design, with each participant responding to one random stimulus from each of five modal categories in a randomized order, resulting in five stimuli in total. After each stimulus participants were asked to confirm a chills response, and answer questions about their experience. The study was approved by the Institutional Department of Music Ethics Committee, written informed consent was obtained from all participants, and the use of Reddit was in compliance with their user agreement and content policies as of September 24, 2018. Complete raw participant sample descriptives, full datasets, and stimulus sets analysed for this study are openly available in a data repository at Harvard Dataverse [70], and in the supplementary materials (S1 and S2 Tables).

### Participants

A total of 179 participants took part in the study, of which 114 were female, 52 were male, five were transgender or other, and eight did not provide gender data. The mean age of the sample was 30.91 (SD = 11.20, range = 18–74). The majority of the sample was comprised of students of different ages and levels of education (52%), although a variety of occupations were reported (administration = 10%; healthcare and nursing = 9%; teaching = 6%; management = 6%). Numerous different nationalities were also represented in the sample (British and United Kingdom = 51%; German = 8%; American = 6%; Canadian = 3%), and participants listened to the study audio through several modes (computer speakers = 54%; headphones = 32%; desk speakers = 3%; unspecified = 11%). On a scale of one (*very high proficiency*) to five (*very low proficiency*), all participants reported being proficient with English language and comprehension (M = 1.31, SD = 0.54), with no participant reporting less than a moderate proficiency level (no ratings higher than three).

Participants were invited to take part in the study through social media outlets, such as Facebook, Twitter and institutional mailing lists; participants were not recruited through the Reddit ‘frisson’ forum, to reduce levels of familiarity with the stimuli that might confound results. The focus on chills experiences was an explicit part of the study advertisements, which possibly resulted in a specialized sample of the population (i.e. chills responders); this characterisation was further supported through 102 participants reporting that they experience aesthetic chills roughly between monthly and daily in their lives (from a possible five categories of *yearly, every few months, monthly, weekly* and *daily*). However, this sample specification was considered a vital approach to maximise the elicitation of chills, given the current aims of exploring and elucidating distinctions between different chills experiences, and considering the general rarity of the response in empirical settings.

### Stimuli

A total of 15 stimuli were selected for this study, with images, texts, videos, music performance videos (MVs), and music excerpts chosen according to a specific criteria. Firstly, for any music, video or MV stimulus, the item would need to be an appropriate duration for the study, and if not, the stimulus would need to provide a meaningful and representative epoch to be extracted (i.e. a contextualised structure, Mnarrative or topic that is clearly communicated). Stimulus durations were determined through an initial pilot test (N = 11), to validate the chills efficacy of the material. Feedback from this procedure suggested that music, videos and MVs could be of one minute or less and still effectively elicit chills, which was important for reducing fatigue and length of the overall study, especially considering the focus on intense emotional experiences. Furthermore, participants in the initial test noted that as images and texts did not change over time like other stimulus types, presentation times of more than 20 seconds induced boredom, with the chills response often occurring within this timespan. Therefore, music, videos and MVs were presented to participants for between 45 to 60 seconds, judged to be enough time to depict meaningful events or narratives, and control for fatigue; in contrast, images and texts were presented for 20 seconds.

Secondly, the three highest rated stimuli for each modal category on the online forum were to be selected following the suitability check criteria and process. For the musical excerpts, three stimuli were instead chosen from a previous listening experiment on musical chills [20], with excerpts from identified chills sections in these pieces used for the current study (each 56 seconds in duration). Thus, the stimuli ranged from instrumental music and amateur recordings of live performances, to images of war veterans, orphaned gorillas, and videos of scientists observing a successful landing of a rocket (see Table 1). Stimulus modality was not a central question for the current study, as the existing literature, whilst highlighting that chills can occur across many sensory modalities, suggests little reason to anticipate systematic differences in chills experiences across modalities [61, 66]. However, a diversity of modality was crucial to explore extensively, and with more clarity, the effects of stimulus qualities as opposed to modal artefacts on the broader chills response. Consequently, the data-driven thematic qualities and characteristics or the stimuli, and their impact on different chills experiences, was the focus.

**Table 1:** The 15 stimuli used in the study; the *thematic category* labels are derived from a data-driven, agglomerative hierarchical cluster analysis (see Data Analysis and Results sections).

Stimulus	Modality	Thematic Category	Description
**Funeral Haka**	Video	*Communion*	School students performing the Haka at teacher’s funeral procession
**Dog Reunion**	Video	*Love and Gratitude*	Dog and owner reunited after long separation
**Scientists**	Video	*Communion*	Scientists celebrate landing of rocket
**Jupiter**	Music	*Solo Voice or Instrument*	Famous string theme from Gustav Holst
**Ancestral**	Music	*Solo Voice or Instrument*	Guitar solo from Steven Wilson
**Glósóli**	Music	*NA*	Large crescendo from Sigur Rós
**Queen Audience**	Music Video	*Communion*	Audience sings along to recording of Bohemian Rhapsody
**Swiss Band**	Music Video	*Communion*	Fans sing to chorus of song, to performers’ surprise
**Ave Maria**	Music Video	*Solo Voice or Instrument*	Man sings in empty shipping container
**War Veteran**	Image	*Distress and Support*	Older man in distress walks alone during war memorial
**Innocent Man**	Image	*Distress and Support*	Man judged to be innocent after years of imprisonment
**Gorilla**	Image	*Distress and Support*	Orphaned gorilla comforted by person
**Father Pride**	Text	*Love and Gratitude*	Loving message from Father to son
**Professor**	Text	*Love and Gratitude*	Quote from professor about caring for yourself
**Addict**	Text	*Love and Gratitude*	Grateful message from recovering addict to paramedics

### Materials

Self-reports formed the primary source of data for the current study. After each stimulus, participants were asked to confirm whether they experienced something they would describe as chills (yes/no/unsure), and how familiar they were with the stimulus (Likert scale of 1 to 5, reflecting low to high familiarity); additionally, participants could confirm the experience of specific bodily activities with either yes or no answers (change in breathing, cold, frowning, goosebumps, laughter, lump in the throat, shivers, smiling, tears, tingling, warmth and warmth in the chest), and provide various emotional ratings on Likert scales of 1 to 7 (affection, anger, calm, energetic, happy, inspired, intensity, melancholy, moved, nervous, nostalgia, relaxed, sadness, stimulated and tender). Bodily activity and emotion rating selections were motivated by findings in existing research [13, 40, 44, 46, 66, 71].

Once participants had been exposed to the five stimuli randomly selected, the Interpersonal Reactivity Index (IRI) was completed, to assess relationships between trait empathy and the different experiences of chills. This instrument contains 28 items answered on 5-point Likert scales that range from ‘does not describe me well’ to ‘describes me very well’. The psychometric structure of the instrument is comprised of four sub-scales or factors, labelled fantasy, empathic concern, perspective taking and personal distress. The instrument has been widely used and tested for reliability, internal consistency and validity [59, 72], and has been shown to be applicable across different languages [73, 74]. In the current study, the IRI instrument and sub-scales showed good internal consistency comparable to previous research (Cronbach’s alpha: fantasy = .76; empathic concern = .82; perspective taking = .78; personal distress = .79; full IRI scale = .71). The web-study was administered via the Qualtrics platform.

### Procedure

Participants were first presented with an information screen documenting the procedure of the study, followed by an opportunity to provide informed consent. Following this, participants were made familiar with the tasks in the study by presenting a practice image (a Cathedral); the task was to watch or listen to the stimuli presented to them, and to rate their physical and emotional experience afterwards. Once participants were happy to continue, they proceeded through five stimuli, one from each modal category. After the fifth and final stimulus, participants completed the IRI instrument. The study concluded by debriefing participants on the main hypothesis regarding distinct types of chills experiences, and participants were offered the opportunity to be placed into a study raffle, with a chance of winning one £50 Amazon gift voucher. Importantly, due to the focus on how individuals conceptualise the chills response, limitations in existing research regarding the theoretical underpinnings and psychological construct of chills, and proposed differences in experience between goosebumps and shivers [66], participants were not provided with a working definition of the phenomenon until the study had concluded; this was to ensure that participants were not artificially biased towards reporting any specific conceptualisation of chills, allowing the study to explore meaningful, informative variations in how respondents characterise and report the phenomenon. The study lasted approximately 15 minutes.

### Data analysis

Data analysis was performed in R. The IRI instrument scores were totalled to provide an overall trait empathy rating, and also aggregated into the four underlying factors of the instrument. Bodily activity data were treated primarily as count data, coded with 1 for bodily activity experienced, and 0 for no activity experienced; furthermore, mean ratings for subjective feeling data were calculated, and alongside bodily activity responses, formed the main level of analysis. To assess the overall frequency of chills across modal categories of stimuli, Poisson regression was utilised to account for positively skewed, repeated-measures data. To analyse chills frequency data within each stimulus modality, chi-square tests were performed, given that each participant responded to only one stimulus from each modal category, meeting assumptions of independence.

To assess the first experimental hypothesis (H1) related to possible distinct categories of chills experiences, an exploratory analysis strategy was devised to reduce bodily activity and subjective feelings to fewer, coherent components. Firstly, data were filtered to include only self-reported confirmed experiences of chills across the stimuli. If a participant reported no chills to a certain stimulus or was unsure as to whether chills were experienced, this response was removed from the analysis; this was a crucial decision to avoid conflating chills experiences with other unrelated affective responses, allowing for a more robust methodology and interpretability of results. Next, reports of bodily activity were chosen as the starting point for identifying differing components across chills responses; due to the count data and repeated-measures design, the method of multiple correspondence analysis (MCA) was utilized through the R package ‘*FactoMineR*’ [75], with bodily activity as the main dependent variable. This method develops dimensions that best describe the variance in the data, with highly explanatory dimensions maintained for analysis and interpretation. An important note is that eigenvalues generated from MCA are generally smaller than more traditional factor analysis approaches, therefore as opposed to retaining eigenvalues of a value greater than one [76], Greenacre [77] suggests that dimensions can be retained if they correspond to eigenvalues equal to or above one divided by the number of variables in question. Output from MCA highlights which bodily responses are best represented by the main dimensions, and produces eta^2^ correlations between the dimensions and the data, with statistical significance determined by calculating *z*-test statistics; this results in preliminary categories and groupings in bodily activity data. In addition, MCA allows for the designation of supplementary variables, such as demographic or participant data; in this case, the subjective feeling rating scales were utilized as supplementary variables, to provide a preliminary visualization and interpretation of the relationships between the bodily activity dimensions and emotional responses. Following this process, identified bodily activity groupings were correlated with emotional descriptors through polyserial correlations; the outcome of assessing these relationships would be finalized distinct chills categories comprised of bodily activity and subjective feeling responses. Whilst the method is similar conceptually to principle components analysis, MCA is optimized to work with categorical, binary dependent variables, and repeated-measures designs [78].

To develop average scores for different chills categories, bodily activity data were first aggregated and converted to numerical data (e.g. for any specific chills category: no corresponding bodily activity = 0, experiencing two corresponding responses = 2); next, both bodily activity and emotion rating ranges were standardized from 1 to 5, with chills category scores calculated by averaging over the corresponding bodily activity and emotion data groupings developed through the MCA and polyserial correlations.

Finally, to investigate relationships between these chills categories and stimulus characteristics, data-driven stimulus themes and distinctions were established through an agglomerative hierarchical cluster analysis of the overall chills category ratings across individual stimuli; this utilised a Euclidean distance similarity matrix of the mean chills category scores. From these themes, a confirmatory analysis strategy was then employed, assessing the effects of stimulus themes (H2) and individual differences (H3) on the experience of different chills categories. To achieve this, linear mixed effects models were fitted using the ‘*lme4*’ R package [79], with chills category scores as the dependent variable, stimulus themes and trait empathy fitted as fixed effects, and both participants and individual stimuli included as random effects; this method was selected to control for variance across individual approaches to the study, and for idiosyncrasies within specific stimuli. Statistical significance was established through likelihood ratio tests, by comparing the full statistical model with a reduced comparative model. This procedure was carried out three times for each chills category: Firstly, to assess the contribution of stimulus theme to the model (H2), secondly to assess the contribution of trait empathy to the model (H3), and thirdly to assess the possible interactions between stimulus theme and empathy. As an extended assessment of these models, vuong tests were carried out using the R package ‘*nonnest2*’ [80], to check that the models were sufficiently distinguishable for the tests.

## Results

### Descriptive statistics

Across 179 participants experiencing 5 stimuli each, a total of 344 chills responses were reported, meaning that chills occurred in roughly 38% of experiences; given the rarity of chills responses, this suggests that the stimulus selection process was reasonably effective for a sample of chills responders, although it is important to note that this result is likely not indicative of chills prevalence across a broader population. With regards to the IRI trait empathy scores, mean total score out of a maximum of 140 was 89.35 (SD = 9.86); no significant differences between males (M = 87.20, SD = 11.72) and females (M = 90.04, SD = 8.88) were found for trait empathy (*t* = -1.51, *df* = 73.56, *p* = .13); additionally, no differences were found across the four trait empathy sub-scales (fantasy: *t* = -1.33, *df* = 72.44, *p* = .18; empathic concern: *t* = -1.13, *df* = 72.47, *p* = .26; perspective taking: *t* = -0.97, *df* = 71.32, *p* = .33; personal distress: *t* = -.184, *df* = 85.68, *p* = .06). Results showed that participants were mostly unfamiliar with the stimuli presented (M = 1.97, SD = 1.28, range = 1–5).

### Frequency of chills

To explore the general efficacy of various stimuli in eliciting the chills response, the frequency of chills experiences reported was first analysed across the modal category level (image, text, video, music, MV). The video category resulted in 99 chills responses, followed by 72 chills with MVs, 66 responses with images, 57 experiences with music, and 50 reports of chills with texts. Regarding differences in chills frequency across modalities, results showed that the only significant effect was found for videos (*β* = 0.40, *SE* = 0.15, *z* = 2.55, *p* = .01), suggesting that whilst images, texts, music and MVs elicited similar frequencies of chills, videos were more effective.

Extending the analysis, the frequency of chills was assessed within each stimulus modality, to see if specific stimuli were particularly effective at eliciting chills. Results highlight that only the music category showed a significant effect of individual stimulus (*x*^2^ = 13.61, *df* = 2, *p* = .001), with Jupiter (chills N = 31) being a more effective elicitor in the study, as opposed to Glósóli (N = 12) and Ancestral (N = 14).

Regarding gender, it was found that females reported more chills (N = 238) than males (N = 88); however, the sample was largely comprised of female participants, and Holm corrected comparisons suggested that this difference was not significant (*β* = 0.19, *SE* = 0.13, *z* = 1.47, *p* = .28).

### Distinct chills experiences

To assess the first experimental hypothesis (H1), an exploratory analysis strategy was utilised; MCA was carried out across chills reports, with reported bodily activity as the main dependent variable, and subjective feeling ratings as supplementary variables. By visualizing the variances explained by dimensions using a scree plot and utilising Greenacre’s eigenvalue considerations [77], three dimensions were initially retained for analysis constructed from bodily activity data: the first dimension explained 16.23% of variance (eigenvalue = 0.16), the second dimension explained 12.68% of variance (eigenvalue = 0.13), and the third dimension explained 11.75% of variance (eigenvalue = 0.12); the total explained variance was 40.67%. For these dimensions, the eta^2^ values are presented in Table 2. Results show that for the first dimension, significant medium strength correlations were found for frowning, smiling, feelings of warmth, and feelings of cold; for the second dimension, significant, medium strength correlations were found for tingling, shivers and goosebumps; finally, for the third dimension, significant, medium strength correlations were found tears and feeling a lump in the throat. It was anticipated that shivers and goosebumps might correlate strongly with differently valenced dimensions [66]; however, the second dimension was primarily characterized by bodily activity encapsulated by traditional conceptualizations of chills (goosebumps, shivers, tingling), a dimension that was judged to be unrevealing due to data being filtered to contain only chills experiences. For this reason, the first and third MCA dimensions formed the main source of interpretation. The first dimension was primarily characterized by coldness and frowning on one end of the axis, and smiling and warmth on the other; the third dimension was characterized mainly be the feeling of a lump in the throat, or experiencing tears during chills.

**Table 2:** Eta^2^ values for bodily activity data on the first three dimensions of the multiple correspondence analysis (medium to large correlations are marked in bold).

Response	Dimension 1	Dimension 2	Dimension 3
Smile	.**51**	.01	.02
Warmth	.**36**	.02	.00
Cold	.**29**	.01	.05
Frown	.**28**	.01	.06
Warmth in chest	.23	.00	.07
Goosebumps	.04	.**49**	.00
Shivers	.00	.**41**	.03
Tingling	.01	.**39**	.00
Lump in throat	.00	.00	.**57**
Tears	.00	.08	.**39**
Laughter	.12	.05	.04
Breathing	.05	.00	.14

**Fig 1:**
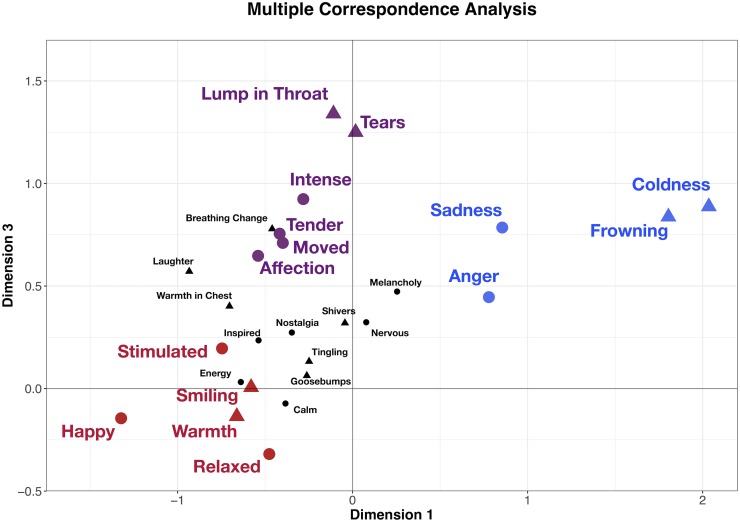
Visualization of the Multiple Correspondence Analysis. Shapes indicate physical (triangle) and emotional (circle) responses, and colours indicate chills categories developed through MCA and polyserial correlations (red = *warm chills*, blue = *cold chills*, purple = *moving chills*).

Supplementary variables comprised of emotion data were also plotted onto the first and third dimensions developed in the MCA (Fig 1), suggesting that the first dimension was characterized by ratings of sadness, anger, happiness, energy and stimulation; the third dimension appeared to be characterized by ratings of being moved, tenderness, affection and emotional intensity. To assess the statistical relationship between bodily activity and subjective feelings, polyserial correlations were carried out between physical activities significantly correlated with the first and third MCA dimensions, and each emotional descriptor (Table 3). Results showed that for warmth and smiling responses, significant medium to strong correlations were found with happy, stimulated, and relaxed ratings; for coldness and frowning, significant medium to strong correlations were found with sadness and anger; finally, for lump in the throat and tears, significant medium correlations were found with affection, tenderness, being moved, and emotional intensity.

**Table 3:** Polyserial correlations between main bodily activity variables from MCA, and emotional descriptors (medium to large positive correlations in bold text).

Response	Smile	Warmth	Cold	Frown	Lump in throat	Tears
Happy	.**72**	.**50**	-.54	-.64	-.08	-.04
Stimulated	.**31**	.28	-.14	-.36	.05	-.03
Relaxed	.24	.**36**	-.13	-.19	-.16	-.25
Sadness	-.64	-.28	.**44**	.**51**	.**40**	.**40**
Anger	-.41	-.21	.**52**	.**67**	.20	.14
Affection	.14	.**40**	-.11	-.11	.**33**	.**41**
Tender	.09	.28	-.01	.02	.**37**	.**37**
Moved	.02	.**31**	-.12	-.08	.**39**	.**50**
Intensity	-.02	.16	.03	.02	.**42**	.**50**
Energetic	.**34**	.17	-.09	-.24	-.08	-.08
Calm	.16	.28	-.16	-.13	-.01	-.06
Inspired	.16	.24	-.22	-.22	.13	.07
Melancholy	-.25	-.08	.21	.21	.20	.17
Nostalgia	.003	.13	-.10	-.27	.10	.08
Nervous	-.14	-.09	.25	.10	.02	.06

Following the MCA and polyserial correlations, final distinct chills categories were constructed, characterized by groupings in, and between, both bodily activity and emotional experience data: *warm chills* (warmth, smiling, happiness, stimulated and relaxed), *cold chills* (coldness, frowning, sadness and anger), and *moving chills* (lump in the throat, tears, affection, tenderness, being moved and intensity). These aggregated, mean chills category ratings were not significantly different across gender or familiarity ratings; furthermore, it is worth noting that when adopting a more inclusive data analysis approach (i.e. retaining ‘unsure’ responses from participants regarding chills), comparable results are found.

### Distinct chills, stimulus themes and empathy

**Fig 2:**
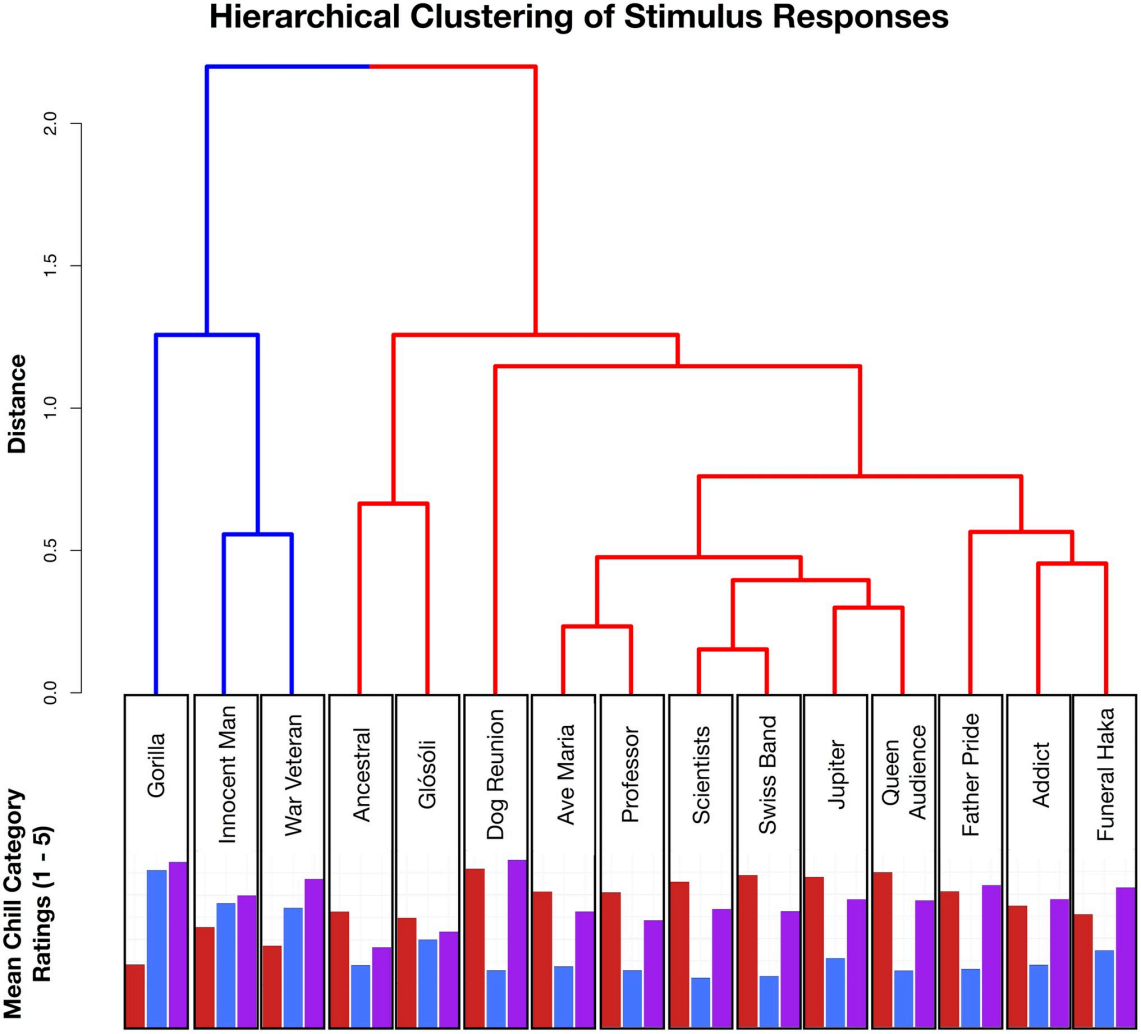
Full solution dendrogram from the agglomerative hierarchical cluster analysis. Red and blue branch colours indicate the two cluster solution representing positive and negative valence; smaller bar graphs indicate raw mean ratings for *warm* (red), *cold* (blue) and *moving* (purple) chills for each individual stimulus.

In assessing the second (H2) and third (H3) experimental hypotheses, namely the effects of stimulus themes and individual differences on warm, cold and moving chills, an exploratory analysis strategy was utilised, followed by a confirmatory analysis. First, an agglomerative hierarchical cluster analysis was performed on chills category ratings across the individual stimuli; by silhouette plotting and dendrograms, the optimal number of clusters to retain was two, which together portray a distinction between negative and positive valence across the stimuli (see Fig 2). By assessing the qualitative similarities between stimuli, the negative cluster stimuli could be categorized thematically in terms of expressions of *distress and support* (visible distress, injustice, support from one to another); the positive cluster stimuli could be characterized by three thematic qualities, namely *communion* (moments of group motor or vocal synchrony), *love and gratitude* (affection and messages of love), and *solo voice or instrument* (solo vocal performance, guitar solo or string theme). The excerpt Glósóli could not be thematically categorized, and was omitted from further analysis. Stimuli were henceforth organized by the author into these thematic categories (see Table 1); this was to accommodate the unbalanced number of negative and positive stimuli, and to increase the level of resolution concerning stimulus qualities and corresponding effects, derived from the data-driven positive and negative two cluster solution.

For the confirmatory analyses, linear mixed effects models were fitted to data for each of the three chills categories, to assess effects of stimulus theme and individual differences. Regarding effects of stimulus theme (H2), results for warm chills showed that stimulus theme had a significant effect on experience (*x*^2^ = 18.05, *df* = 3, *p* <.001). Holm corrected post-hoc comparisons showed that the theme of distress and support was rated significantly lower for warm chills compared to communion (*β* = -1.40, *SE* = 0.25, *z* = -5.55, *p* <.001), love and gratitude (*β* = 1.30, *SE* = 0.25, *z* = 5.06, *p* <.001), and solo voices and instruments (*β* = 1.21, *SE* = 0.27, *z* = 4.43, *p* = .008). For cold chills, model comparisons showed a significant effect of stimulus theme on experience (*x*^2^ = 31.27, *df* = 3, *p* <.001). Corrected post-hoc comparisons suggested that this effect of theme was driven by distress and support, which received significantly higher cold chills ratings when compared to communion (*β* = 1.67, *SE* = 0.17, *z* = 9.35, *p* <.001), love and gratitude (*β* = -1.70, *SE* = 0.18, *z* = -9.37, *p* <.001), and solo voices and instruments (*β* = -1.58, *SE* = 0.19, *z* = -8.16, *p* <.001). Finally, results for moving chills showed that stimulus themes had a significant effect on experience (*x*^2^ = 8.54, *df* = 3, *p* = .03); however, corrected post-hoc comparisons revealed only one significant difference between themes, with distress and support resulting in higher moving chills ratings, compared to solo voices and instruments (*β* = -0.88, *SE* = 0.27, *z* = -3.13, *p* = .01). It is worth noting that carrying out these hypothesis tests at the level of positive or negative clusters of stimuli (as opposed to thematic categories) results in highly comparable effects, although no significant effect is found for moving chills (warm chills: *x*^2^ = 17.41, *df* = 1, *p* <.001; cold chills: *x*^2^ = 30.78, *df* = 1, *p* <.001; moving chills: *x*^2^ = 3.10, *df* = 1, *p* = .07).

In relation to trait empathy (H3), for warm chills there was neither a significant effect of empathy on results (*x*^2^ = 2.42, *df* = 1, *p* = .11), or significant interaction between theme and empathy (*x*^2^ = 2.59, *df* = 3, *p* = .45). Concordantly, for cold chills there was neither a significant effect of empathy (*x*^2^ = 0.55, *df* = 1, *p* = .45), or significant interaction between stimulus theme and empathy for cold chills ratings (*x*^2^ = 3.42, *df* = 3, *p* = .33). For moving chills, as with warm chills and cold chills, there was no significant interaction between stimulus theme and empathy (*x*^2^ = 6.14, *df* = 3, *p* = .10); however, there was a significant effect of empathy on moving chills ratings (*x*^2^ = 4.51, *df* = 1, *p* = .03), with higher empathy scores resulting in higher mean scores for moving chills responses.

Alongside the cluster analysis, these results suggest that whilst warm and cold chills correspond to the positive and negative valence distinction across stimuli, moving chills are experienced similarly across most stimuli, with trait empathy possibly playing an important role in predicting moving chills experiences. In a final assessment of model comparisons, results from the vuong tests suggest that only one comparison involved models that were not significantly distinguishable, mainly the full cold chills model and corresponding no empathy reduced model; whilst there appears to be no clear association between trait empathy and cold chills ratings, this result should be approached with some degree of caution. The likelihood ratio test statistics and post-hoc comparison summaries are presented in Table 4.

**Table 4:** Likelihood ratio tests statistics and post-hoc comparisons, to assess effects of stimulus themes and trait empathy on warm, cold and moving chills (*** = *p* < .001, ** = *p* < .01, * = *p* < .05).

Chills Category	Fixed Effects	Likelihood Ratio (*x*^2^)	Post-Hoc (Theme Comparisons)	z-statistic
**Warm Chills**	*Stimulus Theme*	18.05***	Distress and Support < Communion	-5.55***
			Love and Gratitude > Distress and Support	5.06***
			Solo Voice and Instrument > Distress and Support	4.43**
	*Trait Empathy*	2.42		
**Cold Chills**	*Stimulus Theme*	31.27***	Distress and Support > Communion	9.35***
			Love and Gratitude < Distress and Support	-9.37***
			Solo Voice and Instrument < Distress and Support	-8.16***
	*Trait Empathy*	0.55		
**Moving Chills**	*Stimulus Theme*	8.54*	Solo Voice and Instrument < Distress and Support	-3.13*
	*Trait Empathy*	4.51*		

## Discussion

The current study aimed to assess the possibility of distinct chills experiences, as proposed by previous research [10, 13, 62, 63, 66], and to further elucidate the possible underlying processes of the responses through analysing differences across stimuli. The identification of distinct categories of experience within the chills construct has important ramifications for existing research, and future investigations.

The present study highlighted three categories of chills responses. Firstly, *warm chills* were experiences accompanied by positively valenced feelings such as joy, stimulation and relaxation, and bodily activity such as smiling and feelings of warmth. These experiences were linked to stimuli depicting instances of social communion, such as music audiences singing together or scientists celebrating together, and loving scenarios, including reunions between pet and owner or a message of pride from father to son. Secondly, *cold chills* were experiences accompanied by negatively valenced feelings such as sadness and anger, and bodily activity such as frowning and feelings of cold. Cold chills appear to be best elicited by stimuli that depict an instance of injustice, distress, and visible support from one person to another person or animal in need. Finally, *moving chills* were accompanied by bodily activity such as tears and a lump in the throat, and were characterized mainly by feelings of tenderness, affection, intensity, and being moved. These experiences appeared to be elicited frequently across most stimuli, but are the only responses significantly related to levels of trait empathy. These findings suggest that there exist at least three separable chills constructs, distinguished based on affective valence, qualities of the elicitors, and individual differences. Furthermore, it is inferred from the online forum source of the stimuli utilized, that both warm and cold chills are experiences pursued and sought after by a specific population, regardless of affective valence; however, future research should consider in more detail the hedonic experience of differing kinds of chills experiences, perhaps through the replication of previous neuroimaging work [9].

There are important implications for this preliminary distinction between warm and cold chills, particularly for research into states of being moved. Warm and cold chills appear to align with a distinction in the being moved construct between joyfully and sadly moving scenarios and experiences [16, 32, 33]. In earlier work, Tokaji [33] demonstrated that the same film clip could be sadly or joyfully moving, depending on the context provided; more recently, Wassiliwizky et al. [16] suggested that joyfully moving experiences may be elicited by film clips in which a positive foreground event takes place within an overarching negative context (e.g. reunion after a long separation), and that sadly moving responses are linked to negative events within a positive context (e.g. self-sacrifice to save one’s family). Notably, a stimulus involving a reunion between dog and owner was strongly associated with positive, warm chills responses, indicating states of being joyfully moved; in contrast, a distressed war veteran marching alone in what appeared to be an anniversary parade, conveying new loneliness as the last surviving veteran, was a highly effective elicitor of cold chills responses. The significance of a phenomenological distinction between joyfully and sadly moving experiences is in the psychological processes underlying these responses, with additional implications for being moved and intensified communal sharing relations (CSRs) [44]. Being moved may be elicited by observing the intensification of CSRs in others (third-person), by experiencing a CSR with another person or entity (second-person), or even through memories and internal processes related to CSRs (first-person); furthermore, the intensification of CSRs may be a result of many different processes, with *shared experiences* or *empathic concern* both portrayed in the stimuli used in the current study, and both linked to warm and cold chills respectively. For example, expressions of shared experiences in stimuli such as a large audience singing and moving to music, scientists celebrating together, or school students performing a Haka at their teacher’s funeral, consistently resulted in reports of warm chills experiences and responses, which may be linked to processes related to social identity, belonging, bonding and closeness. In contrast, stimuli expressing themes of distress, emotional discomfort, and a display of comfort or support being offered from one person to another person or animal, seem linked to empathic concern, a process previously associated with goosebumps, tears and warmth in the chest [57], and presently to cold chills. In other words, warm chills may reflect joyfully moving scenarios, most effectively elicited by instances of shared experiences, whereas cold chills reflect sadly moving scenarios, consistently elicited by events that more readily invite empathic concern. This more detailed distinction suggests that whilst CSRs offer a broad psychological mechanism for various states of being moved, kama muta, and related emotional experiences such as nostalgia and elevation [40, 43, 44], it is crucial to develop an understanding of the qualitative differences regarding how exactly CSRs are intensified, at what level, and how these differences may result in meaningful, phenomenological distinctions in states of being moved and related chills responses.

Although it is intuitive to distinguish between warm and cold chills in the current study, the third, moving chills category appears to be more complex. Moving chills were mainly categorized by intense bodily experiences such as tears and feeling a lump in the throat, emotional intensity, and feelings of affection, tenderness and being moved. The response appears to reflect a more prototypical conceptualization of being moved, a seemingly bittersweet, mixed emotional response with social underpinnings (i.e. tears with feelings of affection). The response was associated with most of the stimuli in the current study, although moving chills were also linked to differences in trait empathy across participants. Whilst one interpretation of the analysis could be that moving chills do not denote a separate category of chills, this does not appear to be the case, given that their bodily activity and subjective feeling responses grouped distinctly from other ratings, and that moving chills were the only experiences to be predicted by individual differences. The finding that moving chills were relatively prevalent across most stimuli further supports the interpretation of warm and cold chills as reflecting positive and negative nuances of broader being moved experiences linked to socially or personally significant life events [32, 34, 35]. As trait empathy seemed important only for the moving chills category, this suggests that there is a significant role of individual differences in the experience of certain kinds of chills responses, with phenomenological aspects of chills potentially mediated by empathy. This is a highly pertinent point for existing and future research on individual differences; for example, musical chills have been linked to openness to experience [67–69], and familiarity with the music [3, 81], but in the context of distinct chills responses, these differences may not mediate or predict chills responses at the holistic level of incidence as previously reported. Instead, individual differences may influence the phenomenological qualities and type of chills experienced, and influence which distinct psychological mechanisms may more commonly underlie the experience for a particular person. When considering the theoretical distinction between fear or vigilance and social processes underlying chills, these individual differences may be contextualized, especially with music, through a corresponding empathizing-systemizing distinction in listeners [82]. In this distinction, it is suggested that systemizers tend to process formal structures, regularities, patterns and rule systems; in contrast, empathizers tend to identify emotions in other people or stimuli, and respond in emotionally appropriate ways. By extension, if some individuals prefer to process music at the level of structure, pattern, repetition and syntax, then the aesthetic and emotional experience may tend to be derived aspects such as dynamic changes, crescendos, expectancy violations or unprepared harmonies, all possibly linked to fear and vigilance chills responses [5, 11, 22]. On the other hand, a listener may prefer to process music in terms of narrative [83], social relationships [84], and personas or characters [85]; instead, aesthetic and emotional experiences might be derived from solo instruments, lyrics and the human voice, all aspects also linked to musical chills [12, 13].

Whilst conjecture, the distinction between different chills experiences and the role of individual differences has three important implications for existing aesthetic chills research. Firstly, if individual differences in cognitive processing (possibly linked to aspects such as openness to experience and empathy) influence the tendency to experience a certain type of chills response, then claims about the prevalence of chills with music (and potentially other aesthetic engagements) are misleading in current research, especially given the overuse of classical music repertoire that may place an emphasis on structural aspects in music as opposed to empathic or social aspects. Secondly, and consequently, there may be a notable number of musical features linked to chills that have been undetected in previous research, with some correlations and relationships being over-represented, and others unreported; this is potentially becoming evident through a recent survey [13], which reported the previously undocumented, widely-reported effectiveness of lyrics and the human voice in eliciting chills across a representative sample of listeners. Finally, the current lack of a clear conceptualization of the chills response restricts the development of research aiming to test causal processes and hypotheses linked to theories of the phenomenon; without moving beyond correlations to test causal relationships, the evolutionary and psychological antecedents of aesthetic chills cannot be discerned. Recent work has made an initial step beyond correlations in the field of music and emotion [20], but for a methodical, empirically grounded research agenda to be developed regarding aesthetic chills, future investigations should focus on refining the conceptualization of the chills response, to guide and contextualize the causal testing of existing theories currently proposed for the phenomenon.

There are numerous limitations in the current study that are worth highlighting. Firstly, the web-study paradigm may have introduced confounds such as variations in concentration, attention and experience that were not possible to measure. Similarly, no objective measures of emotional experience were collected, meaning that the temporal correspondence between chills reactions and self-reported experiences could not be fully elucidated. However, the web-study was considered to be the optimal method for developing investigations into distinct chills experiences, especially given the lack of understanding, evidence, and prior research that currently restricts more experimental, psychophysiological endeavours. Secondly, there is the issue of allowing participants to approach chills with individualized conceptualizations of the response. Given that the conceptualization of chills was a central question of the current study, it was not advisable to provide a working definition to participants before the study, as would normally be the case. It is possible that participants reported chills experiences beyond the ‘traditional’ working definition, such as crying or feeling other sensations as opposed to goosebumps, shivers or tingling; on the other hand, given that there exists little research into the details and variations in what people describe as chills, attention should be given to the ways in which people discuss these phenomenological responses. In other words, whilst there is a risk of increasing the range of descriptors and aspects of a chills experience to the scope of inconveniencing experimental approaches, variation in emotional responses was the focus of the study, with numerous implications for existing and future research, and the theoretical discourse underlying chills. Thirdly, when theoretically framing aesthetic chills in relation to evolutionary functions such as thermoregulation, it is important to note that it remains unclear how the chills phenomenon resembles or relates to mammalian goosebumps reactions, nor is it fully understood how engagements with multimedia are associated with thermoregulatory responses; further conceptual work would help to clarify how to situate aesthetic chills, for example by considering the role of goosebumps or shivers as symptoms in fevers and seizures [86, 87]. Finally, whilst different modalities were included in this novel investigation to enrich and extensively explore chills experiences, possible modality-specific effects were not controlled, and given the rating system of the web-forum used to identify suitable stimuli, some modalities, such as the images, were comprised only of negative themes and content. Although there is no immediate reason to suspect clear effects of modality on chills experiences [61, 66], future systematic work on the topic could utilise more stringent control measures for modality, possibly by ensuring a balance and equivalence of thematic quality or affective composition of stimuli across modalities.

In conclusion, the current study provides the first investigation into the possibility of distinct chills experiences in response to various aesthetic stimuli and multimedia. Results suggest that preliminary chills categories may be distinguished by reports of bodily activity and subjective feeling, with warm and cold chills differentiated mainly in terms of affective valence qualities, and possibly underlying processes of shared experience and empathic concern respectively; this distinction reflects being joyfully and sadly moved, and supports the need for further detailed research into experiences of being moved. Furthermore, the moving chills category appears to be prevalent across all stimuli used in the current study, but this seemingly more intense emotional response was the only category linked to higher trait empathy in participants. This third chills category and its relation to empathy highlights the importance of individual differences in aesthetic chills; furthermore, the findings suggest that the contextualization of individual difference effects should be understood not in terms of whether chills are experienced or not in response to an aesthetic stimulus, but in terms of the tendency to experience certain kinds of chills over others. There are numerous avenues for future research with regards to aesthetic chills, such as exploring and elucidating the variety of chills responses in aesthetic engagements, such as awe-related states [88], testing existing fear and social theories of aesthetic chills, and extending studies of individual differences through assessing the effects of personality, preference and empathy on the variations of chills responses reported. Finally, it is highly important to build on the initial chills distinctions presented in the current study, and to attempt to measure objective differences in chills responses that may contribute to understanding the psychological and evolutionary antecedents of chills experiences in aesthetic contexts.

## Supporting information

S1 Table Number of chills responses to the fifteen multimedia stimuli, ordered by relative efficacy. (DOCX)

S2 Table Descriptive statistics for the participant sample. Values in parentheses denote standard deviations.
(DOCX)
