# Supplementary material for: Distinct varieties of aesthetic chills in response to multimedia
Source: PLoS One. 2019 Nov 14;14(11):e0224974. doi: 10.1371/journal.pone.0224974 (PMC6855651; doi:10.1371/journal.pone.0224974)
Supplement: S1 Table — (DOCX) [file pone.0224974.s001.docx]

**S1 Table**: Number of chills responses to the fifteen multimedia stimuli, ordered by relative efficacy.

| **Stimulus** | **Modality** | **Thematic Category** | **Chills Frequency** | **Source** |
| --- | --- | --- | --- | --- |
| Funeral Haka | Video | *Communion* | **34** | https://www.youtube.com/watch?v=M6Qtc_zlGhc |
| Dog Reunion | Video | *Love and Gratitude* | **33** | http://i.imgur.com/IEHbOVc.gifv |
| Scientists Celebrate | Video | *Communion* | **32** | https://www.youtube.com/watch?v=igEWYbnoHc4 |
| Jupiter | Music | *Solo Voice or Instrument* | **31** | Taken from album ‘*The Planets / Suite de Ballet, op. 10*’ (Released 1991 under Naxos) Catalogue number = 8.550193 Barcode = 4891030501935  Musicbrainz Identifier = 2e5015c8-c0a1-4b50-aa68- 2dad4529c972 |
| Queen Singalong | Music Video | *Communion* | **28** | https://www.youtube.com/watch?v =cZnBNuqqz5g&feature=youtu.be |
| Ave Maria | Music Video | *Solo Voice or Instrument* | **27** | https://www.youtube.com/watch?v=a04O1RmCS8M |
| War Veteran | Image | *Distress and Support* | **26** | https://imgur.com/MyxObav |
| Innocent Man | Image | *Distress and Support* | **21** | https://imgur.com/3tMeMSu |
| Father Pride | Text | *Love and Gratitude* | **21** | https://www.reddit.com/r/Frisson/ comments/5kd1ks/text_a_bit_unconventional _for_this_sub_but_one_of/ |
| Gorilla | Image | *Distress and Support* | **19** | https://i.imgur.com/Y01CTLO.jpg |
| Professor Quote | Text | *Love and Gratitude* | **19** | http://i.imgur.com/ZAgoWG6.jpg |
| Swiss Band | Music Video | *Communion* | **17** | https://www.youtube.com/watch?v =IEng60LouQo&feature=youtu.be |
| Ancestral | Music | *Solo Voice or Instrument* | **14** | Taken from album ‘*Hand. Cannot. Erase.*’ (Released 2015 under Kscope) Catalogue number = KSCOPE316 Barcode = 802644831671  Musicbrainz Identifier = 0cfad70c-4fa9-43c2-918e- eb638f0dd597 |
| Glósóli | Music | *NA* | **12** | Taken from album ‘*Takk...*’ (Released 2009 under EMI Music Australia) Catalogue number = 695 4682 Barcode = 5099969546822  Musicbrainz Identifier = d79744e1-a616-3e75-844d- 9d83ad5da6a3 |
| Recovered Addict | Text | *Love and Gratitude* | **10** | https://www.reddit.com/r/Frisson/ comments/52w7xq/text_a_thank_you_letter_from _a_heroin_addict/ |
