# Supplementary material for: Distinct varieties of aesthetic chills in response to multimedia
Source: PLoS One. 2019 Nov 14;14(11):e0224974. doi: 10.1371/journal.pone.0224974 (PMC6855651; doi:10.1371/journal.pone.0224974)
Supplement: S2 Table — Values in parentheses denote standard deviations. (DOCX) [file pone.0224974.s002.docx]

**S2 Table**: Descriptive Statistics for the Participant Sample; values in parentheses denote standard deviations.

| Variable | Male | Female | Transgender / Other / Unreported | **Overall** |
| --- | --- | --- | --- | --- |
| **Sample Size** | 52 | 114 | 13 | **179** |
| **Mean Age** | 32.31 (10.05) | 30.37 (11.89) | 28.83 (6.46) | **30.91 (11.20)** |
| **Overall Chills** | 88 | 238 | 18 | **344** |
| **Mean Chills** | 1.69 | 2.08 | 1.38 | **1.71** |
| *Image Chills* | 17 | 45 | 4 | **66** |
| *Video Chills* | 25 | 69 | 5 | **99** |
| *MV Chills* | 16 | 52 | 4 | **72** |
| *Text Chills* | 15 | 34 | 1 | **50** |
| *Music Chills* | 15 | 38 | 4 | **57** |
| **Chills Type (Aggregate Rating)** |  |  |  |  |
| *Warm Chills* | 2.84 (1.03) | 2.90 (1.07) | 2.83 (1.00) | **2.88 (1.05)** |
| *Cold Chills* | 1.71 (0.88) | 1.67 (0.92) | 1.75 (0.86) | **1.69 (0.90)** |
| *Moving Chills* | 2.74 (0.90) | 3.00 (1.04) | 2.82 (0.90) | **2.95 (1.00)** |
| **IRI Mean** | 87.20 (11.72) | 90.04 (8.88) | 94.16 (8.32) | **89.35 (9.86)** |
| *Fantasy* | 22 (4.95) | 23.01 (3.53) | 25.33 (2.80) | **22.79 (3.97)** |
| *Empathic Concern* | 21.60 (3.45) | 22.21 (2.48) | 23.33 (1.50) | **22.07 (2.79)** |
| *Perspective Taking* | 22.88 (4.29) | 23.53 (3.02) | 24.66 (4.63) | **23.37 (3.49)** |
| *Personal Distress* | 20.13 (4.07) | 21.35 (3.53) | 20.83 (3.31) | **20.97 (3.72)** |
| **Play an Instrument** |  |  |  |  |
| *Yes* | 27 | 39 | 3 | **69** |
| *No* | 25 | 75 | 2 | **103** |
| **General Chills Frequency** |  |  |  |  |
| *Yearly* | 5 | 10 | 0 | **15** |
| *Every few months* | 14 | 23 | 0 | **37** |
| *Monthly* | 7 | 19 | 1 | **27** |
| *Weekly* | 10 | 24 | 3 | **48** |
| *Daily* | 7 | 20 | 0 | **27** |
| **Musical Sophistication** | 2.49 (1.06) | 2.24 (0.85) | 2.80 (0.83) | **2.33 (0.92)** |
| **English Proficiency** | 1.34 (0.59) | 1.31 (0.53) | 1.00 (0) | **1.31 (0.54)** |

**Note:** ‘overall chills’ describes the number of total chills reported, whereas ‘mean chills’ indicates the average number across the subsamples (to accommodate different subsample sizes). English proficiency was rated on a scale of 1 (*very high proficiency*) to 5 (*very low proficient*). Musical sophistication was rated on a scale of 1 (*non-musician*) to 6 (*professional musician*).
